# The Emerging Threat of Monkeypox: An Updated Overview

**DOI:** 10.3390/v18010069

**Published:** 2026-01-03

**Authors:** Galal Yahya, Nashwa H. Mohamed, Al-Hassan Soliman Wadan, Esteban M. Castro, Amira Kamel, Ahmed A. Abdelmoaty, Maha E. Alsadik, Luis Martinez-Sobrido, Ahmed Mostafa

**Affiliations:** 1 Department of Microbiology and Immunology, Faculty of Pharmacy, Zagazig University, Al Sharqia 44519, Egypt; galalyehia@zu.edu.eg; 2 Molecular Biology Institute of Barcelona (IBMB), Spanish National Research Council (CSIC), 08028 Barcelona, Spain; 3 Hospitals of Zagazig University, Zagazig 44519, Egypt; nashwahashem28@gmail.com; 4 Food Hygiene, Safety and Technology Department, Faculty of Veterinary Medicine, Zagazig University, Zagazig 44519, Egypt; 5 Oral Biology Department, Faculty of Dentistry, Galala University, Galala Plateau, Attaka 15888, Egypt; al-hassan.mohamed@gu.edu.eg; 6 Host-Pathogen Interactions (HPI) and Disease Intervention and Prevention (DIP) Programs, Texas Biomedical Research Institute, San Antonio, TX 78227, USA; emcastro@txbiomed.org; 7 Dermatology, Venereology and Andrology Department, Zagazig University Hospitals, Faculty of Medicine, Zagazig University, Zagazig 44519, Egypt; amirakamel1799@gmail.com; 8 Internal Medicine Department, Faculty of Medicine, Mutah University, Karak 61710, Jordan; drahmedatya33@gmail.com (A.A.A.); mahaalsadik@gmail.com (M.E.A.); 9 Department of Gastroenterology, Hepatology and Infectious Disease, Faculty of Medicine, Zagazig University, Zagazig 44519, Egypt; 10 Chest Department, Faculty of Medicine, Zagazig University, Zagazig 44519, Egypt; 11 Center of Scientific Excellence for Influenza Viruses, National Research Centre, Giza 12622, Egypt

**Keywords:** monkeypox, orthopoxvirus, zoonosis, antivirals, vaccines, climate changes, global warming

## Abstract

Monkeypox (MPOX) is an emerging zoonotic disease caused by monkeypox virus (MPXV), an orthopoxvirus closely related to smallpox. Initially confined to endemic regions in Central and West Africa, MPOX has recently gained global significance with outbreaks reported across multiple continents. MPXV is maintained in animal reservoirs but is increasingly transmitted from person to person, facilitated by close contact, respiratory droplets, and, in some cases, sexual transmission. Clinically, MPOX presents with fever, lymphadenopathy, and a characteristic vesiculopustular rash, though atypical manifestations have been observed in recent outbreaks, complicating diagnosis. Laboratory confirmation relies on molecular testing, while differential diagnosis must consider varicella, herpes, and other vesicular illnesses. Therapeutic options remain limited; supportive care is the cornerstone of management, but antivirals such as tecovirimat and brincidofovir, as well as smallpox vaccines, have shown efficacy in mitigating disease severity and preventing infection. The unprecedented global outbreak has underscored the importance of surveillance, rapid diagnostics, and coordinated public health responses to contain transmission. This review provides an overview of epidemiology, virology, clinical manifestations, modes of transmission, available diagnostics, and prophylactic and therapeutic strategies against MPOX. We also discuss the role of animal reservoirs, viral evolution, and human-to-human transmission in shaping the dynamics of recent MPOX outbreaks. By summarizing the latest evidence, this review aims to inform clinicians, researchers, and policymakers about key aspects of MPOX biology, clinical management, and prevention, while identifying gaps that warrant future investigation for the control of this and potentially other emerging zoonotic-related pathogens with an impact on human health.

## 1. Introduction

Monkeypox (MPOX) is a zoonotic infection caused by monkeypox virus (MPXV) that belongs to the Orthopoxvirus genus, which includes other notable viruses such as variola virus (VARV) that causes smallpox, vaccinia virus (VACV) that is used in the smallpox vaccine, and cowpox virus (CPXV) (Table 1) [1]. Since the eradication of smallpox in 1980, other poxviruses like CPXV and MPXV have received increased attention due to their ability to cause sporadic epidemics [2]. Human infections with CPXV occurred recently in Europe and Western Asia and are associated with infected cattle and domestic cats as a source of CPXV [3,4]. In addition, multiple strains of VACV are currently circulating in South America, where they cause outbreaks in dairy cattle and pose an occupational risk to dairy handlers [5,6]. Nevertheless, the global spread and increased risk of MPOX since 2022 warrant focused discussion.

MPOX disease was first identified in 1958 when outbreaks of a pox-like disease occurred in monkeys kept for research purposes, hence the name “monkeypox”. However, MPXV is primarily found in rodents and other small mammals in the rainforests of Central and West Africa, which are considered possible natural reservoirs [7]. Human cases of MPOX were first identified in 1970 in the Democratic Republic of Congo (DRC), and since then, sporadic outbreaks have been reported in various parts of Africa. Recently, there has been an increase in MPOX cases outside Africa, raising concerns about its potential to become a global public health issue [8].

**Table 1:** Comparison of various poxviruses [9,10].

Aspect	Monkeypox Virus	Variola (Smallpox) Virus	Cowpox Virus	Vaccinia Virus
Abbreviation	MPXV	VARV	CPXV	VACV
Genome Size	~196–211 kb	~186 kb	~220 kb	~196 kb
Gene Count	~190 genes	~187 genes	~223 genes	~200 genes

## 2. MPXV Infection and Pathogenesis

### 2.1. MPXV Structure and Genome Composition

**Figure 1:**
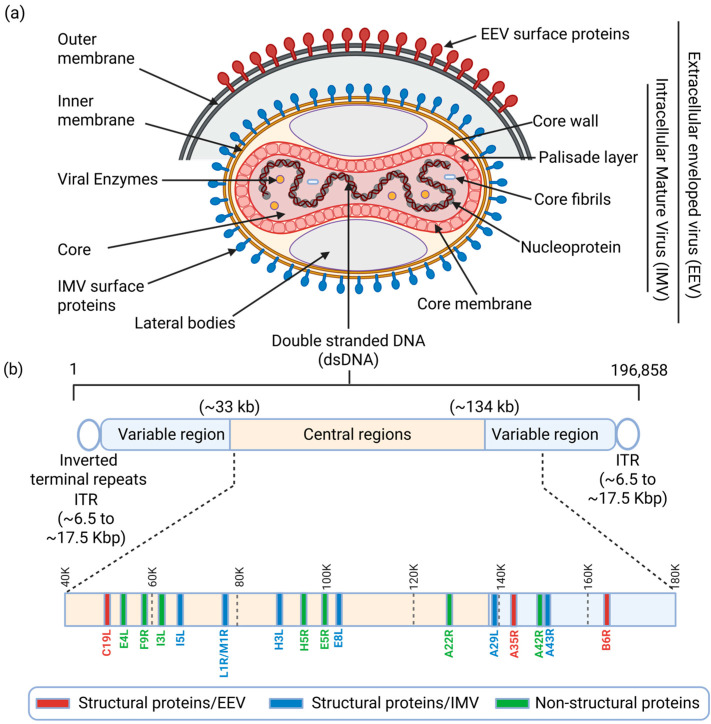
Schematic representation of the extracellular enveloped virus (EEV) and the intracellular mature virion (IMV) of MPXV and viral genome/protein organization. (**a**) The structure of the EEV and IMV contains similar distinct structures, including the inner membrane and surface tubules/viral proteins, lateral bodies, palisade layer, core and core fibril, and viral dsDNA, whereas the EEV has an extra, fragile outer membrane with additional viral proteins. (**b**) The MPXV genome is composed of two terminal variable regions with inverted terminal repeat (ITR, hairpin loops, ~6.5 kb to ~17.5 kb) and a large conserved central genomic region (~33 kb to ~134 kb). The approximate coding regions of the “housekeeping” structural proteins of the EEV (red) and IMV (blue) and non-structural proteins involved in viral replication, transcription and assembly (green) are mainly encoded in the conserved central genomic region. The figure has been assembled and created using biorender.com.

MPXV is a large, ovoid or brick-shaped virus that measures approximately 200–250 nm in diameter [11]. It is an enveloped double-stranded (ds)DNA virus with a genome length of approximately 197.2 kb, containing more than 190 open reading frames (ORFs) that code for various proteins involved in viral replication, host immune evasion, and pathogenesis (Table 1) [12]. The MPXV replication cycle includes viral particle attachment to host cell surface glycosaminoglycan (GAG) receptors like chondroitin sulphate and heparan sulphate, fusion, viral genome replication, virion assembly, and release from the infected host cell [13]. Meanwhile, two types of infectious forms of MPXV are produced: extracellular enveloped virus (EEV) and intracellular mature virus (IMV) (Figure 1a). The EEV is secreted via exocytosis and consists of an intracellular IMV particle enclosed by an additional outer lipid membrane derived from the Golgi apparatus or endosomes [13]. The EEV form of MPXV displays several surface membrane proteins, including C19L, A35R, and B6R (Figure 1a), whereas the IMV form is released during cell lysis and presents several key viral surface proteins such as A29L, A43R, H3L, E8L, I5L and L1R/M1R (Figure 1b) that play critical role in the formation and stabilization of the lipoprotein envelope [13]. This stabilized IMV form makes the MPXV suitable for transmission between animals, whereas the EEV form spreads better from cell to cell within the same host [14]. IMVs enter neighboring host cells through macropinocytosis, whereas EEVs enter host cells via fusion [15].

The non-structural proteins that include A22R, A42R, E4L, E5R, F9R, H5R and I3L, are not part of the virion itself (Figure 1b) [14]. However, they play essential roles in immune evasion, viral replication and assembly. As crucial mediators of the viral life cycle, these non-structural proteins perform distinct essential functions: A42R initiates viral genome replication; A22R is integrated into the viral core to form the replication machinery [14]; E5R enhances replication via interaction with host proteins [14]; F9R facilitates the necessary replication complex formation [14]; H5R regulates the overall viral life cycle, likely through interaction with cellular machinery [14]; and I3L contributes to both viral assembly and the complete replication cycle [14]. Furthermore, E4L promotes viral success by actively inhibiting host immune responses, specifically by interfering with the interferon (IFN) signaling pathway [14].

The viral central core contains the dsDNA genome and core fibrils, which are tightly encircled by the tight “palisade layer”, a highly organized, protein-based lattice forming the boundary of the viral core (Figure 1b). The viral genome is composed of a central conserved region that is flanked by two variable terminal domains (Figure 1b) [11]. A key feature of this genome is the presence of inverted terminal repeats (ITRs), which span 6.5 to 17.5 kb at each end [16]. To stabilize the viral genome, the ITRs form covalently closed ends (hairpin loops), meaning it lacks typical free 3′ and 5′ termini (Figure 1b). The central conserved region encodes the viral proteins required for viral transcription, replication, and virion assembly [11,12], while the genes of the terminal domains vary between different poxviruses and encode proteins involved in host–virus interactions [11,12] (Figure 1b).

### 2.2. MPXV Genotypes/Phenotypes and Epidemiological Relevance

**Figure 2:**
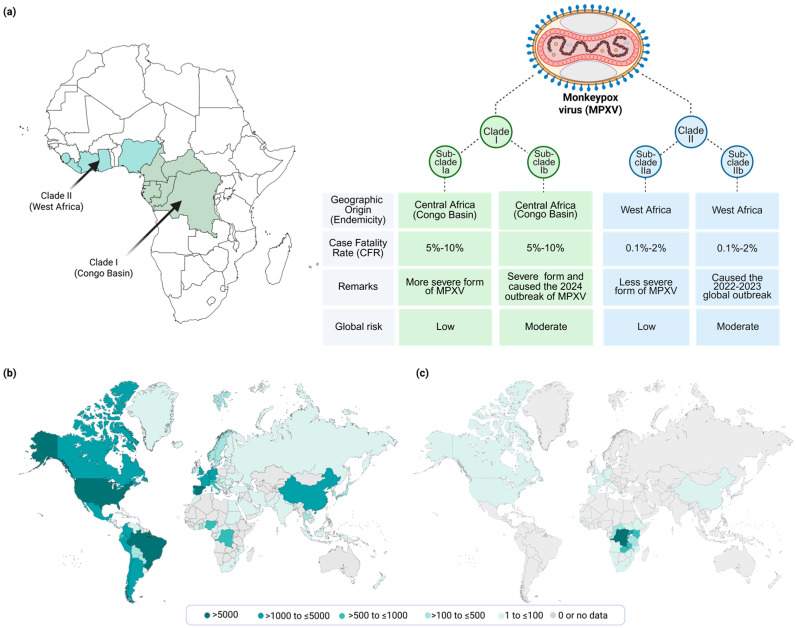
Epidemiology of MPXV. (**a**) Schematic representation of different MPXV clades, subclades, geographic location, and their role in previous and current MPOX outbreaks. (**b**) The 2022–2023 global MPXV outbreak caused by subclade IIb. Data presented as of January 2022 to October 2023 were obtained from Global.health (https://map.monkeypox.global.health/country; accessed on 1 December 2025). (**c**) The 2024–2025 global MPXV outbreak caused by subclade Ib. Data presented from January 2024 to November 2025 were obtained from the WHO [21]. The map was assembled and created using biorender.com.

Since the discovery of the first human infection with MPXV in the DRC, cases have been predominantly reported in rural and rainforest regions of the Congo Basin in DRC, its neighboring countries, and in West Africa, with occasional exported cases reported outside Africa linked to travel or animal importation from endemic regions [17,18]. In 2022, a major epidemiological shift occurred following the occurrence of a global outbreak with extensive human-to-human transmission across more than 100 non-endemic countries, resulting in more than 100,000 confirmed human cases (Figure 2a) [19]. Despite the fact that all MPXV cases were linked to international travel or African animal imports, the 2022 MPXV global outbreak was uniquely characterized by efficient human-to-human transmission following direct contact with infectious sores or lesions on mucous membranes, indicating genetic, geographic, and phenotypic diversification of the virus [17,20].

Historically, MPXV are genetically divided into two major clades: clade I, previously known as the Congo Basin or Central African clade, and clade II, previously known as the West African clade [22,23]. Recent surveillance data from DRC in 2023 led to the identification of a genetically distinct lineage within clade I, now designated as subclade Ib, while all earlier phylogenetic analyses had already delineated multiple lineages within clade I, currently grouped as clade Ia [24,25,26]. Similarly, clade II is further subdivided into subclades IIa and IIb, with subclade IIb being responsible for the 2022 global outbreak (Figure 2b) [26]. In 2023, a new variant of MPXV clade I was first reported in the DRC, namely subclade Ib. In 2024, this variant elevated the virus to a significant global health concern, leading the World Health Organization (WHO) to classify it as a public health emergency. The Ib variant exhibits increased transmissibility, especially through human-to-human and sexual contact. Originating in the DRC, clade Ib has spread swiftly to adjacent African nations and has now been detected in Europe (e.g., Belgium, Germany, the United Kingdom [UK], Italy, France and Spain), Asia (e.g., China, Qatar and United Arab Emirates) and North America (e.g., Canada and the United States [USA]) (Figure 2c) [26]. Since 2024, cases reported in DRC, Congo, Mozambique, and Senegal are known to be a mix of subclade Ib and/or subclade Ia, and/or subclades IIa/b [21].

Clades I and II can cause MPXV infection in humans; however, infections with clade II are generally milder and exhibit lower transmissibility compared to those caused by clade I [27,28,29]. Notably, within MPXV clades, clade I isolates have a more uniform genome length (196 Kbp–199 Kbp) than clade II isolates (196–211 kilobases, kb) [1]. Clade I and II MPXV genomes differ by ≈0.4–0.5% in nonrepetitive regions conserved between the clades and by the presence of 4 large insertions/deletions [22].

### 2.3. MPXV Life Cycle

**Figure 3:**
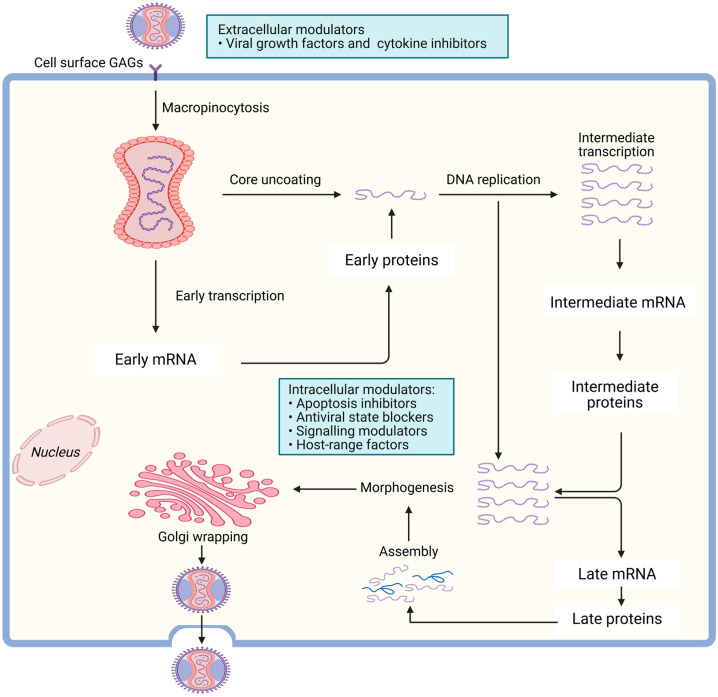
Replication cycle of MPXV. MPXV infection begins with viral entry and fusion via macropinocytosis, following viral particle binding to host cell surface GAGs [31]. Following internalization and core uncoating, early transcription generates early mRNAs, which are translated into early proteins that initiate DNA replication. Intracellular modulators, including apoptosis inhibitors, antiviral state blockers, signaling modulators, and host-range factors, enhance viral survival and replication. Intermediate transcription produces intermediate mRNAs and proteins, which drive genome replication and morphogenesis. Late transcription yields late mRNAs and proteins that are essential for virion assembly. Mature virions undergo Golgi wrapping before being released from the host cell, completing the replication cycle. The figure has been assembled and created using biorender.com.

The life cycle of MPXV is similar to that of other Orthopoxviruses and occurs entirely within the cytoplasm of the host cell (Figure 3) [30]. The virus enters host cells via macropinocytosis or direct fusion with the cell membrane, mediated by viral surface proteins that interact with host cell receptors. Once inside, the virus releases its core into the cytoplasm, where early genes are transcribed and translated to produce proteins that help the virus take over the host cell’s machinery [9]. The viral DNA is then uncoated, allowing replication of the viral genome and the production of late-stage proteins required for viral assembly. Mature virions are assembled in the cytoplasm, followed by morphogenesis, and then released from the cell either by cell lysis in the case of IMV or by budding off with part of the host cell membrane in the case of EEV, which helps the virus evade the immune system (Figure 3).

### 2.4. MPXV Immune Evasion Mechanisms

**Figure 4:**
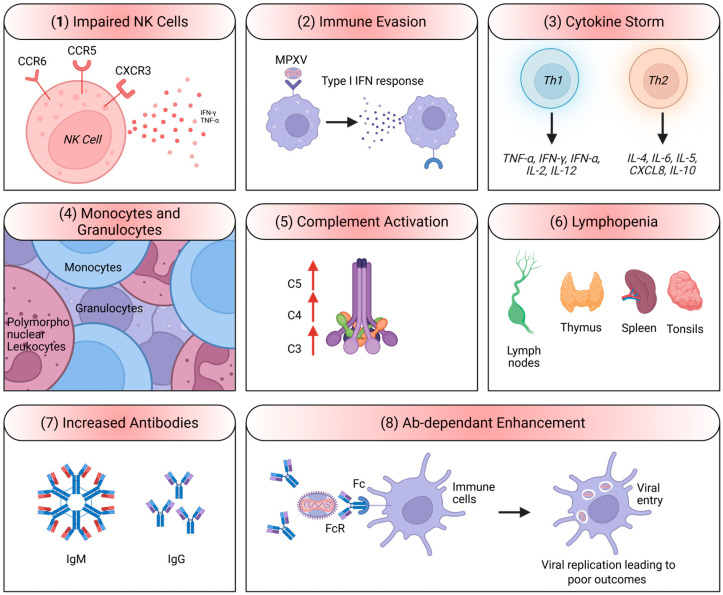
Host immune responses and dysregulation during MPXV infection. (**1**) Natural killer (NK) cell activity is impaired due to altered expression of chemokine receptors (CCR5, CXCR3, CCR6), reducing production of IFN-γ and TNF-α. (**2**) MPXV evades immune detection by suppressing type I IFN (IFN-α and IFN-β) responses. (**3**) A cytokine storm occurs, characterized by upregulation of Th1 cytokines (IFN-α, IFN-γ, TNF-α, IL-2, IL-12) and Th2 cytokines (IL-4, IL-6, IL-5, CXCL8, IL-10). (**4**) Abnormalities in circulating monocytes and granulocytes contribute to dysregulated immunity. (**5**) Complement activation (C3–C5) promotes inflammation and tissue damage. (**6**) Lymphopenia affects secondary lymphoid tissues including lymph nodes, thymus, spleen, and tonsils. (**7**) Elevated antibody levels (IgM, IgG) develop as part of the adaptive response. (**8**) Antibody(Ab)-dependent enhancement (ADE) facilitates viral entry via Fc receptor–bearing immune cells, potentially worsening disease outcomes. The figure has been assembled and created using biorender.com.

Like other Orthopoxviruses, MPXV develops several mechanisms to evade the host immune system [32]: (1) inhibition of IFN responses, where the virus encodes proteins that inhibit the induction and signaling pathways that lead to the production of IFN responses, which are crucial for the antiviral immune response [33,34]; (2) blocking apoptosis, where MPXV encodes anti-apoptotic proteins that prevent the programmed cell death of infected cells, allowing the virus to replicate for a longer period [35]; (3) modulation of host cytokine responses, where the virus encodes cytokine-like molecules and receptors that can modulate the host’s immune response to create a more favorable environment for viral replication [35]; and (4) MHC class I downregulation, where MPXV interferes with the presentation of viral antigens on MHC class I molecules, reducing the ability of CD8^+^ T cells to recognize viral peptides presented by MHC class I molecules and thereby delay viral clearance by CD8^+^ T cells [36,37] (Figure 4). Host immune responses against MPXV infection are marked by significant dysregulation, exemplified by NK cell function impairment through altered chemokine receptor signaling, leading to reduced IFN-γ and TNF-α production [38]. MPXV further evades host defenses by suppressing type I IFN (IFN-α and IFN-β) pathways, facilitating viral persistence [35]. A dysregulated cytokine profile, with simultaneous upregulation of Th1 and Th2 cytokines, contributes to an exaggerated inflammatory response resembling a cytokine storm [39]. Hematological abnormalities, including altered monocyte and granulocyte activity and marked lymphopenia affecting secondary lymphoid tissues, reflect widespread immune disruption [38]. Complement activation (C3–C5) enhances inflammation, while elevated IgM and IgG responses indicate activation of the adaptive immune system [40]. However, antibody(Ab)-dependent enhancement (ADE) has been proposed as a mechanism that may worsen disease by promoting viral entry into Fc receptor–bearing cells [41]. Collectively, these findings suggest that MPXV pathogenesis is driven not only by viral replication but also by an imbalance of the host immune responses (Figure 4) [33,34].

## 3. MPXV Epidemiology and Outbreaks

### 3.1. Historical Context and Endemic Regions

MPOX has been primarily endemic in Central and West Africa, with the DRC being the most affected country [42]. MPXV was first isolated from a nine-month-old male child in the DRC in 1970, suspected of having smallpox [43]. Since then, the disease has been reported sporadically, with major outbreaks occurring in the DRC, Nigeria, and surrounding countries [1,44,45]. The incidence of MPOX in Africa has been increasing over the past few decades [44]. Multiple factors contribute to the rise in MPXV. First, waning smallpox immunity, following the eradication of smallpox and the cessation of mass vaccination programs in the early 1980s, resulted in MPXV immunity waning in the population, leading to increased susceptibility [46]. Environmental changes, such as deforestation, urbanization, and human encroachment into wildlife habitats, have increased human exposure to MPXV reservoirs [47]. Finally, increased human mobility from improved transportation infrastructure and greater movement between rural and urban areas facilitate the spread of the virus to more populated regions [48].

### 3.2. Recent MPOX Outbreaks and Global Spread

In recent years, MPOX has attracted international attention due to outbreaks in non-endemic regions outside of Africa [49]. Notably, in 2003, the United States of America (USA) experienced its first MPOX outbreak, which was linked to imported Gambian pouched rats that infected prairie dogs sold as pets [50]. This outbreak resulted in 47 confirmed and probable cases but no fatalities [51]. In 2017, Nigeria experienced a significant resurgence of MPOX after nearly 40 years without reported cases [52]. This outbreak has persisted, with ongoing cases reported annually. The Nigerian outbreak raised concerns due to its spread to urban areas and the potential for international transmission [44]. In 2022, MPOX cases were reported in several non-endemic countries, including the USA, Canada, the UK, and several European Union (EU) countries. These cases were primarily associated with human-to-human transmission, including transmission among men who have sex with men (MSM), highlighting the virus’s potential for spread in new populations [17,20]. In 2025, an imported MPXV strain of clade Ia was identified and genomically characterized after being isolated from a traveler returning from the DRC to China [53]. In contrast, several subclade Ib MPOX cases were reported in Spain, Italy, Portugal, and the Netherlands with no travel history, indicating autochthonous transmission of MPXV subclade Ib in the EU through sexual networks among MSM [54].

## 4. MPXV Clinical Presentation and Symptoms

### 4.1. Incubation Period and Initial Symptoms

The incubation period for MPXV typically ranges from 6 to 13 days but can extend up to 21 days [55]. During this period, the virus replicates within the host without causing noticeable symptoms [55]. The initial clinical presentation is often non-specific, resembling other viral infections [56,57,58,59]. The early symptoms of MPOX include fever as one of the earliest and most common symptoms, severe headache, generalized muscle pain and discomfort, significant back pain, and swelling of lymph nodes, particularly in the neck, armpits, and groin (lymphadenopathy) [60]. Lymphadenopathy is a distinguishing feature that helps differentiate MPOX from smallpox [61]. Patients often experience severe fatigue and a general sense of malaise [60].

### 4.2. Rash Development and Progression

After the initial symptoms, a rash typically develops within 1 to 3 days following the onset of fever. The rash often begins on the face and then spreads to other parts of the body, including the palms of the hands, soles of the feet, and mucous membranes. The rash progresses through five stages [62,63,64]: (1) Macules: Flat, red spots appear on the skin. (2) Papules: The macules become raised, forming papules. (3) Vesicles: the papules fill with a clear fluid, forming vesicles. (4) Pustules: The vesicles become deep-seated, firm pustules, filled with a thick, opaque fluid. (5) Scabs: Eventually, the pustules crust over and form scabs, which will later fall off and possibly leave scars that may be permanent. The rash typically resolves within 2 to 4 weeks, and patients are considered contagious until all scabs have fallen off.

### 4.3. Severity of Symptoms

**Figure 5:**
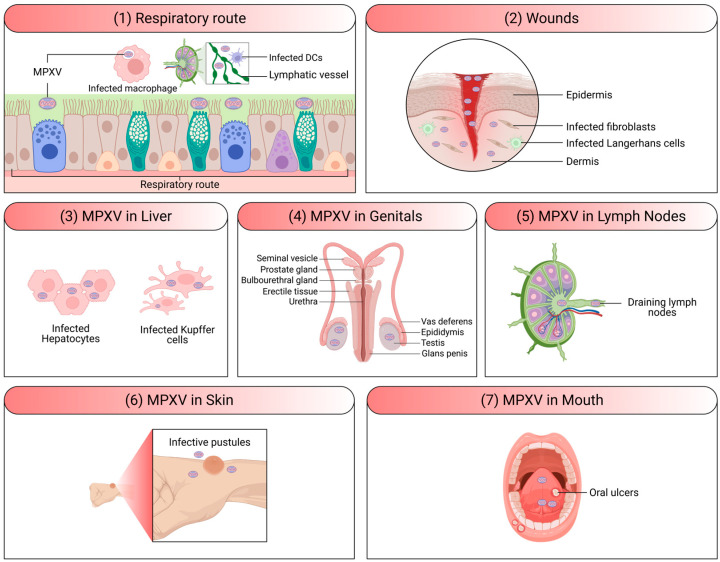
Tropism and clinical manifestations of MPXV. (**1**) Respiratory route: The respiratory tract serves as a major entry route, where MPXV infects dendritic cells (DCs) and macrophages that subsequently migrate via lymphatic vessels. (**2**) Wounds: MPXV entry through skin wounds involves infection of fibroblasts and Langerhans cells in the epidermis and dermis. (**3**) MPXV can be detected in hepatocytes and Kupffer cells of the liver. (**4**) MPXV has also been identified in the genital area [73,74], raising the possibility of sexual transmission. (**5**) Infected macrophages and NK cells accumulate in draining lymph nodes. (**6**) Clinically, MPOX manifests as multiple pustular skin lesions resulting from MPXV infection of epidermal and dermal cells. (**7**) Extensive oral ulcerations may develop, reflecting mucocutaneous involvement. The figure has been assembled and created using biorender.com.

While MPOX is generally a self-limited disease, the severity of symptoms can vary (Figure 5) [15]. Most patients experience mild to moderate symptoms and recover without the need for medical intervention [65]. However, severe cases can occur, particularly in vulnerable populations such as children, pregnant women, immunocompromised individuals, and those with underlying health conditions [66,67,68]. Complications can include secondary bacterial infections, bronchopneumonia, sepsis, encephalitis, and infection of the cornea, which can lead to vision loss [68,69,70,71]. The case fatality rate for MPOX has historically been reported to be between 1% and 10%, depending on the strain and the population affected. The Central African (Congo Basin) clade I of the virus tends to cause more severe disease with higher mortality rates compared to the West African clade II (Figure 2) [72].

## 5. MPXV Transmission to Humans

### 5.1. Animal-to-Human Transmission

**Figure 6:**
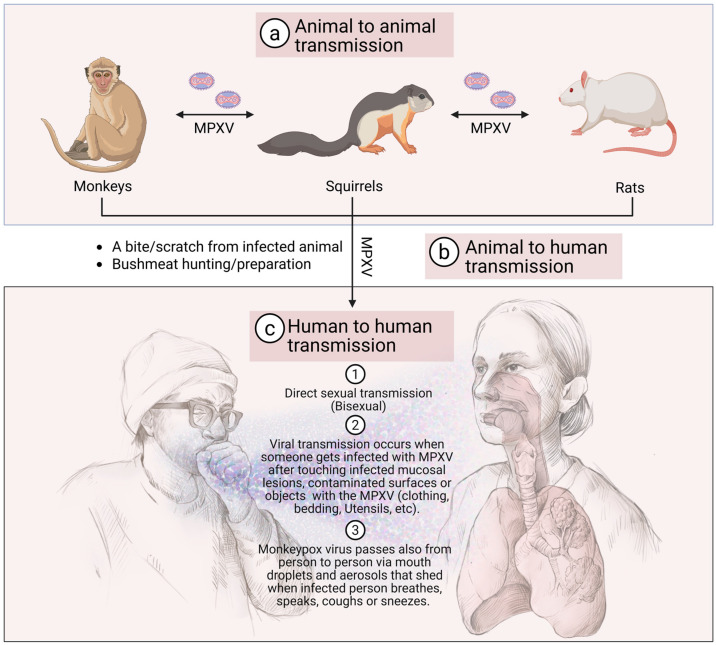
Routes and mechanisms of MPXV transmission. (**a**) Animal-to-animal transmission: MPXV can circulate among different animal species, including non-human primates, rodents, and other mammals. (**b**) Zoonotic transmission (animal-to-human): Humans can acquire MPXV through direct contact with infected animals, their secretions or hunting/preparing bushmeat. (**c**) Human-to-human transmission: Several mechanisms contribute to the spread of MPXV between people, including (1) transmission among men who have sex with men (MSM) through close and intimate contact; (2) direct contact with contaminated mucocutaneous lesions, surfaces, or objects from infected individuals; and (3) transmission via respiratory droplets and aerosols during breathing, coughing, or sneezing, particularly during prolonged face-to-face contact with an infected person. The figure has been assembled and created using biorender.com.

The primary mode of transmission for MPOX is zoonotic, meaning the virus is transmissible from animals to humans [24,75]. Rodents, including squirrels, rats, and mice, are believed to be the main reservoirs of MPXV, though various other small mammals may also harbor the virus [75]. Humans can become infected through direct contact with the blood, bodily fluids, or skin lesions of infected animals. This can occur through (1) bites or scratches from infected animals; (2) handling of infected animal meat, including hunting, skinning, and preparing bushmeat; and/or (3) contact with contaminated surfaces, including bedding, cages, or other materials that have come into contact with an infected animal (Figure 6) [75].

### 5.2. Human-to-Human Transmission

Human-to-human transmission of MPXV occurs primarily through direct contact with an infected person [29]. In recent outbreaks, close physical contact, including sexual contact, has been identified as a significant mode of transmission [76,77]. This has been particularly noted in cases reported among MSM [76]. It is important to note that MPXV was not considered a sexually transmitted disease (STD) in the traditional sense, as it can spread through various forms of close contact [11,78,79]. In addition to entering the body through broken skin (including wounds invisible to the naked eye), the virus can spread largely through fluid or droplets into the mouth, nose, or eyes. These droplets are relatively heavy and often unable to propagate more than a few feet. Therefore, these contaminated respiratory droplets generated during prolonged face-to-face contact with infected people, such as that occurring among family members or in healthcare settings, could contribute to human-to-human transmission [80,81]. The virus can also be transmitted through direct contact with bodily fluids or skin lesions of an infected person, as well as through contaminated objects, such as bedding, clothing, and surfaces [11].

## 6. MPXV Risk Factors and Vulnerable Populations

### 6.1. Populations at Higher Risk

Certain populations are at higher risk of contracting MPXV or experiencing MPOX severe disease [82,83]. These include (1) children: younger children, especially those under 8 years old, are at a higher risk of severe disease and complications [84]; (2) pregnant women: pregnant women are at risk of severe outcomes, including fetal infection, miscarriage, and preterm birth, while evidence suggests vertical transmission from mother to fetus [66,85,86]; (3) immunocompromised individuals: those with weakened immune systems, such as people living with HIV, those on immunosuppressive therapies, or individuals with other underlying health conditions, are more susceptible to severe MPXV infections [87]; (4) people with eczema or other skin conditions: individuals with preexisting skin conditions like eczema may experience more extensive and severe rashes, with a higher risk of secondary infections; and (5) healthcare workers: due to their proximity to patients, healthcare workers are at an increased risk, particularly if proper personal protective equipment (PPE) is not used.

### 6.2. Occupational Exposure

Individuals who work with animals, particularly in endemic regions, are at higher risk for zoonotic transmission. This includes hunters, animal handlers, veterinarians, and laboratory personnel working with orthopoxviruses [88,89,90].

## 7. MPOX Diagnosis and Differential Diagnosis

### 7.1. Clinical Diagnosis

MPOX is often diagnosed based on clinical presentation, particularly in endemic areas [91,92]. The characteristic rash, coupled with a history of potential exposure, can provide strong clues for diagnosis. However, due to the overlap in symptoms with other diseases such as chickenpox, smallpox, and other vesiculopustular rashes, laboratory confirmation is essential [93].

### 7.2. Laboratory Diagnosis

Laboratory confirmation of MPOX involves several methods: (1) Polymerase Chain Reaction (PCR): PCR is the preferred diagnostic test due to its high sensitivity and specificity. Samples are typically taken from skin lesions (e.g., vesicles, pustules, or scabs) and tested for viral DNA [94,95,96]. (2) Serology: Serological tests can detect antibodies against MPXV, but they are less commonly used for acute diagnosis and may be more useful for epidemiological studies [92]. (3) Virus isolation: This involves culturing MPXV from clinical specimens, but is less commonly used due to the need for specialized laboratory facilities and biosafety precautions [97,98,99]. (4) Electron Microscopy (EM): EM can be used to visualize the virus, but this method is more commonly used in research settings.

### 7.3. Differential Diagnosis

Differentiating MPOX from other diseases with similar presentations is crucial for appropriate management and public health response [99,100,101]. The main conditions to consider in the differential diagnosis include chickenpox (Varicella), caused by the varicella-zoster virus (VZV), which presents with a similar vesicular rash [102]. However, chickenpox usually lacks the pronounced lymphadenopathy seen in MPOX [100]. Smallpox, although eradicated, was caused by VARV, producing a rash that is similar to MPOX [103]. The key difference is the history of vaccination and the absence of smallpox in the modern era [104]. Measles can present with a maculopapular rash, but it usually starts behind the ears and on the face and does not form vesicles or pustules [105]. Scabies can cause a widespread itchy rash, but it lacks the systemic symptoms of MPOX and is caused by a mite rather than a virus [106]. Bacterial skin infections, most commonly caused by beta-hemolytic streptococci or Staphylococcus aureus, can lead to impetigo or Ecthyma that can cause pustular lesions, but they are typically localized and do not progress through the same stages as MPOX lesions [107].

## 8. MPOX Public Health Impact and Response

### 8.1. Impact on Endemic Regions

In endemic regions, MPOX poses a significant public health burden, particularly in rural and resource-limited settings [108]. The disease can cause outbreaks that strain healthcare systems, particularly in areas where other infectious diseases such as malaria, HIV, and tuberculosis are also prevalent [109,110,111]. The impact extends beyond health, affecting livelihoods, particularly when the disease leads to the culling of livestock or the restriction of hunting and trading of bushmeat [112,113,114,115].

### 8.2. Global Health Concerns

The spread of MPOX to non-endemic regions has raised global health concerns, particularly in light of the coronavirus disease 2019 (COVID-19) pandemic [116]. The potential for MPXV to spread internationally, especially in the context of increased global travel and trade, necessitates a coordinated global response. International public health agencies, such as the WHO and the Centers for Disease Control and Prevention (CDC), have issued guidelines for the surveillance, diagnosis, and management of MPOX [91]. These guidelines emphasize the importance of early detection, contact tracing, isolation of cases, and public health education to prevent and control outbreaks [117,118,119,120,121].

### 8.3. Outbreak Control Measures

Control measures during MPOX outbreaks include surveillance and rapid response [122]. Effective surveillance systems are essential for early detection of cases [122]. Rapid response teams should be deployed to investigate and contain outbreaks. Infected individuals should be isolated to prevent the spread of the virus. Contacts of confirmed cases may be quarantined and monitored for symptoms. The public health authorities must provide clear and accurate information to the public about the risks of MPOX, how it is transmitted, and the steps to take to prevent infection. Eventually, in some settings, vaccination campaigns, where close contacts of confirmed cases are vaccinated, may be employed to contain outbreaks [123,124].

## 9. MPOX Prevention and Protection

### 9.1. Personal Protective Measures

Individuals can take several measures to protect themselves from MPXV, particularly in endemic areas or during outbreaks [125]. Avoid contact with infected animals, which is especially important in areas where the virus is known to circulate among wildlife. Avoiding contact with animals that appear sick or have died of unknown causes is crucial [126]. Safely handling animal products, including proper cooking of meat and wearing protective gear when handling animals or animal products, can reduce the risk of zoonotic transmission [127]. Regular hand washing with soap and water, especially after contact with animals or potentially contaminated materials, is essential [128]. Finally, healthcare workers and others at risk of exposure should use appropriate PPE, including gloves, masks, and eye protection, when caring for patients or handling specimens [129].

### 9.2. Vaccination

Vaccination acts as a crucial first line of defense, including MPOX, by preparing the body to fight pathogens before they can cause serious illness [14]. While the innate immune system provides a non-specific initial defense, vaccines specifically train the adaptive immune system to remember and rapidly defeat targeted pathogens [38]. This preparation is a primary strategy for both individual and community health. Therefore, vaccination plays a crucial role in preventing MPOX, particularly in high-risk populations [130].

#### 9.2.1. Smallpox Vaccination and Cross-Protection

The smallpox vaccine, derived from VACV, has been shown to provide cross-protection against MPXV [131]. Studies have estimated that the smallpox vaccine is about 35–85% effective in preventing MPXV infection [131,132,133]. However, routine smallpox vaccination was discontinued globally after the eradication of smallpox in 1980, leaving subsequent generations susceptible to MPXV [134,135].

#### 9.2.2. New Vaccines

With the reemergence of MPOX, two vaccines have been approved by the Food and Drug Administration (FDA) for the prevention of smallpox and MPOX disease: JYNNEOS and ACAM2000 [136,137,138,139,140]. JYNNEOS (Imvamune or Imvanex) is a live, non-replicating attenuated vaccine made of a weakened VACV that prevents smallpox and MPOX [141,142]. It is particularly recommended for high-risk populations, such as healthcare workers, laboratory personnel, and close contacts of confirmed cases [143,144,145]. JYNNEOS is administered in two doses given 4 weeks apart. However, ACAM2000 is a live, replicating vaccine that is used for smallpox and provides protection against MPXV [142,146]. It is primarily used for military personnel and laboratory workers due to its potential side effects [144,145,147,148,149,150]. ACAM2000 is administered using a multiple-puncture method on the upper arm. Because of being a live replicating virus, ACAM2000 is not recommended for people with underlying immunodeficiency [142,146].

#### 9.2.3. Infection Control in Healthcare Settings

In healthcare settings, strict infection control measures are critical to prevent the spread of MPXV [129]. Patients with suspected or confirmed MPOX should be isolated in negative-pressure rooms, if available, to prevent airborne transmission [151]. Healthcare workers should wear appropriate PPE, including N95 respirators, gowns, gloves, and eye protection, when caring for MPOX patients [152]. Surfaces and materials that have come into contact with infected patients should be thoroughly cleaned and disinfected using USA Environmental Protection Agency (EPA)-registered disinfectants, effective against orthopoxviruses [153].

### 9.3. Treatment Options

#### 9.3.1. Supportive Care

The cornerstone of MPXV treatment is supportive care, which involves managing symptoms and preventing complications [154,155]. Key aspects of supportive care include ensuring adequate fluid intake to prevent dehydration, particularly in children and patients with severe rash or gastrointestinal symptoms [156,157], and pain management using analgesics and antipyretics to manage fever, pain, and discomfort [157,158]. Treatment of secondary infections with antibiotics may be necessary if secondary bacterial infections develop, particularly in cases of extensive skin lesions [157,159]. Nutritional support may also be necessary to ensure adequate nutrition for recovery, especially in children and malnourished patients [157].

#### 9.3.2. Antiviral Therapies

While there is no specific antiviral treatment approved for MPOX, several antivirals developed for smallpox have shown efficacy in treating MPXV infection (Table 2) [160,161]. Tecovirimat (ST-246) is an antiviral drug that inhibits the activity of the viral envelope VP37 protein, preventing the release of mature virions from infected cells [15]. It has been approved for the treatment of smallpox and has shown efficacy in animal models of MPXV infection [162]. Tecovirimat is available under expanded access protocols or compassionate use for treating MPXV. Cidofovir and Brincidofovir are both antiviral agents that inhibit viral DNA polymerase, thereby interfering with viral replication [163]. While Cidofovir has been used in severe cases of MPXV, it is associated with nephrotoxicity [164]. Brincidofovir, a lipid-conjugated derivative of Cidofovir, has improved safety and has been evaluated in MPXV animal models, although clinical data in human cases is limited (Table 2) [160,161,165].

#### 9.3.3. Immunoglobulins

Vaccinia immune globulin (VIG) is a preparation of antibodies derived from individuals who have been vaccinated against smallpox [169]. VIG has been used to treat complications of smallpox vaccination and has a potential use in MPXV, particularly in immunocompromised patients who may not respond well to vaccination [170].

## 10. MPOX Vaccination Strategies and Cross-Protection

### 10.1. Historical Context of Smallpox Vaccination

The global eradication of smallpox in 1980 marked a significant achievement in public health [171]. The widespread use of the smallpox vaccine not only eradicated smallpox but also provided cross-protection against other orthopoxviruses, including MPXV [134]. However, following the cessation of smallpox vaccination, the immunity provided by this vaccine has waned in the general population, leading to increased susceptibility to MPXV infection [172,173,174,175].

### 10.2. Current Vaccination Recommendations

Considering recent MPOX outbreaks, public health authorities have revisited vaccination strategies [176]. Key recommendations include vaccination for groups or individuals at high risk of exposure, such as healthcare workers, laboratory personnel handling orthopoxviruses, and close contacts of confirmed cases [177]. In an outbreak setting, a ring vaccination approach may be employed, where close contacts of confirmed cases and healthcare workers involved in their care are vaccinated to contain the spread of the virus [178]. Pre-exposure vaccination may even be considered for individuals who work with orthopoxviruses or who live in areas with ongoing MPXV transmission [179].

Monkeypox vaccination can be used as a post-exposure prophylaxis (PEP) for individuals with known or suspected exposure to MPXV, as well as for those with risk factors suggesting possible exposure [180]. PEP is most effective when administered within 4 days of exposure, but vaccination given 4–14 days after exposure may still reduce disease severity [180]. Beyond 14 days, vaccination may be considered on a case-by-case basis, particularly for high-risk individuals such as the severely immunocompromised. Individuals with ongoing risk of exposure should be vaccinated regardless of prior exposure, provided they have not developed symptoms [180]. Vaccination after symptom onset, diagnosis, or recovery is not expected to be beneficial because natural infection is thought to confer immunity, although the duration of protection remains uncertain [180].

### 10.3. Cross-Protection with Other Poxviruses

The genetic similarity between orthopoxviruses allows for cross-protection, where immunity to one poxvirus provides partial protection against others [181]. The smallpox vaccine, which contains live-attenuated VACV, is known to confer immunity against MPXV due to this cross-reactivity [181]. This cross-protection is critical in the context of emerging zoonotic diseases, where existing vaccines can be leveraged to provide protection against newly emerging pathogens [182,183,184]. Predicting whether a disease like MPXV will become a pandemic involves the assessment of several key criteria as outlined here.

#### 10.3.1. Human-to-Human Transmission

For MPXV, while it primarily spreads through direct contact with infected animals or people, there is evidence of human-to-human transmission [185,186,187]. However, its transmission is less efficient compared to respiratory viruses like influenza or SARS-CoV-2. The time between exposure to the virus and the onset of symptoms (incubation period) can affect how quickly it spreads. MPXV has an incubation period of 7–14 days, which can allow for more extended transmission periods before symptoms appear [188,189].

#### 10.3.2. Geographic Spread

For a disease to be classified as a pandemic, it must spread beyond its initial geographic area to multiple countries and continents [190]. MPOX, historically confined to Central and West Africa, has seen cases spread to other regions but has not yet reached the widespread global distribution seen in past pandemics [191].

#### 10.3.3. Disease Severity and Impact

MPOX can be severe, especially in immunocompromised individuals, but the mortality rate is relatively low compared to some other infectious diseases [192]. Moreover, a pandemic typically overwhelms healthcare systems. While MPOX can strain resources, it has not yet reached the levels seen with the recent COVID-19 pandemic [193].

#### 10.3.4. Public Health Response and Vaccination Availability

The availability and effectiveness of vaccines can influence the spread of a disease [194]. There are vaccines available for smallpox that also provide protection against MPXV. In an outbreak, vaccination campaigns can help control the spread. Effective public health measures, such as quarantine, isolation, and contact tracing, are also crucial in controlling outbreaks [195,196].

#### 10.3.5. Public Awareness and Compliance

How the public responds to health advisories and practices preventive measures can impact the spread. Awareness and compliance with preventive measures (like avoiding contact with infected individuals) play a key role in controlling outbreaks.

## 11. MPXV Research and Future Directions

### 11.1. Ongoing Research Efforts

Research on MPXV is ongoing, with several key areas of focus. Vaccine development efforts are underway to develop new vaccines that are safer and more effective, particularly for use in immunocompromised populations. Research is also focused on improving the production and accessibility of existing vaccines like JYNNEOS [133]. New antiviral agents are being tested in preclinical and clinical trials for their efficacy against MPXV [197]. Several studies are also exploring combination therapies to improve outcomes in severe cases [198,199]. Epidemiology and surveillance studies are being conducted to better understand the epidemiology of MPXV, including its transmission dynamics, risk factors, and potential animal reservoirs [49,192]. Improved surveillance systems are being developed to detect and respond to outbreaks more quickly. Pathogenesis is being more fully detailed by understanding the molecular mechanisms of the MPXV life cycle and the viral factors that contribute to virulence and immune evasion, which are crucial for developing targeted therapies [9].

### 11.2. Challenges and Opportunities

While significant progress has been made in understanding and managing MPOX, several research gaps related to MPOX, especially the most virulent subclade Ib, still exist [200]. Besides research gaps, several challenges remain, including ensuring global access to vaccines, particularly in endemic regions; improving vaccine production, distribution, and affordability; and strengthening public health infrastructure, particularly in low-resource settings. Addressing these challenges is essential for effective surveillance, diagnosis, and management of MPOX. The ongoing risk of cross-species transmission from wildlife to humans highlights the need for One Health approaches that integrate human, animal, and environmental health to prevent future outbreaks. Public awareness needs to be enhanced by educating the public about MPOX, particularly in non-endemic regions, to allow for early detection and quick responses to outbreaks. Public health campaigns should focus on reducing stigma and misinformation associated with MPOX disease and vaccination.

### 11.3. Future Directions

Looking ahead, several key areas will shape the future of MPOX prevention and control. Expanding global surveillance networks will be critical for early detection of MPOX and other emerging zoonotic diseases. These networks should integrate data from human, animal, and environmental sources to provide a comprehensive picture of disease risk within a One-Health approach. Integrated vaccination strategies will need to combine MPXV vaccination with other public health interventions, such as routine immunization programs, to improve coverage and reduce the burden of the disease. Research needs to be conducted on long-term immunity, including protection provided by vaccination and duration of cross-protection against MPXV, which will be important for informing vaccination policies. Finally, international collaboration will be required to address the threat of MPOX, setting up systems to share data, resources, and expertise. Global initiatives to combat emerging infectious diseases should prioritize MPOX as a key area of focus.

## 12. Conclusions and Perspectives

MPOX, once considered a rare zoonotic disease confined to Central and West Africa, has emerged as a global public health concern due to recent outbreaks in non-endemic regions. The close relationship of MPOX to smallpox, along with the cessation of routine smallpox vaccination, has left a large portion of the global population susceptible to MPXV infection. Effective prevention and control of MPXV require a multi-faceted approach, including robust surveillance systems, targeted vaccination strategies, public health education, and research into new treatments and vaccines. The lessons learned from smallpox eradication provide valuable insights, but the unique challenges posed by MPOX, such as its animal reservoirs and potential for international spread, demand innovative and sustained efforts. As the world continues to manage the COVID-19 pandemic as an established global health concern and prevent seasonal viral infections (e.g., influenza), the emergence of MPOX serves as a reminder of the constant threat posed by zoonotic diseases. Strengthening global health systems, improving preparedness for emerging infectious diseases, and fostering international collaboration are essential to prevent and mitigate future outbreaks of MPOX and potentially other zoonoses.

## Figures and Tables

**Table 2:** Antiviral agents against MPXV, their mechanisms of action, routes of administration and approved uses.

Antiviral Agent	Mechanism of Action	Administration Route	Approved Uses	References
Tecovirimat (known as TPOXX or ST-246)	Inhibition of the viral envelope protein VP37, which is essential for viral release from infected cells. By preventing maturation and release of new viral particles, it reduces the spread of the virus within the host.	Oral tablets or intravenous (IV)	Approved for smallpox; off-label use for MPXV	[166,167]
Brincidofovir (known as Tembexa, CMX001 or HDP-CDV)	Inhibition of viral DNA polymerase, an enzyme crucial for viral DNA replication. This action prevents the virus from multiplying and spreading.	Oral tablets	Approved for smallpox; off-label use for MPXV	[163,168]
Cidofovir	Inhibition of viral DNA polymerase. Generally, it is less preferred than Brincidofovir due to potential nephrotoxicity. Its mechanism of action is similar to Brincidofovir, interfering with viral DNA replication.	Intravenous (IV)	Off-label use for various poxviruses, including MPXV	[163,164]

## Data Availability

No new data were created or analyzed in this study. Data sharing is not applicable to this article.
